# Synthesis and Characterization of Gel Polymer Electrolyte Based on Epoxy Group via Cationic Ring-Open Polymerization for Lithium-Ion Battery

**DOI:** 10.3390/membranes12040439

**Published:** 2022-04-18

**Authors:** Wei Zhang, Taewook Ryu, Sujin Yoon, Lei Jin, Giseok Jang, Wansu Bae, Whangi Kim, Faiz Ahmed, Hohyoun Jang

**Affiliations:** 1 Department of Applied Chemistry, Konkuk University, Chungju 27478, Korea; arno_zw@hotmail.com (W.Z.); gundam0924@naver.com (T.R.); ysj920126@naver.com (S.Y.); jinlei8761@naver.com (L.J.); jws516@naver.com (G.J.); gelp621@naver.com (W.B.); wgkim@kku.ac.kr (W.K.); 2 Grenoble INP, LEPMI, University of Grenoble Alpes, 38000 Grenoble, France; faiz2310@gmail.com

**Keywords:** polymer electrolyte, LiFSI, in situ polymerization, interfacial resistance, ionic conductivity

## Abstract

The polymer electrolytes are considered to be an alternative to liquid electrolytes for lithium-ion batteries because of their high thermal stability, flexibility, and wide applications. However, the polymer electrolytes have low ionic conductivity at room temperature due to the interfacial contact issue and the growing of lithium dendrites between the electrolytes/electrodes. In this study, we prepared gel polymer electrolytes (GPEs) through an in situ thermal-induced cationic ring-opening strategy, using LiFSI as an initiator. As-synthesized GPEs were characterized with a series of technologies. The as-synthesized PNDGE 1.5 presented good thermal stability (up to 150 °C), low glass transition temperature (T_g_ < −40 °C), high ionic conductivity (>10^−4^ S/cm), and good interfacial contact with the cell components and comparable anodic oxidation voltage (4.0 V). In addition, PNGDE 1.5 exhibited a discharge capacity of 131 mAh/g after 50 cycles at 0.2 C and had a 92% level of coulombic efficiency. Herein, these results can contribute to developing of new polymer electrolytes and offer the possibility of good compatibility through the in situ technique for Li-ion batteries.

## 1. Introduction

The materials of the cathode and anode and electrolyte, as vital components of the lithium-ion battery LIB, have been developed over the past few decades. Nowadays, the LIB has been spotlighted as an alternative energy to fossil fuel because of its low self-discharge rate, high operating voltage, long life, and high-energy density and because it has no memory effect [1,2,3]. Traditional liquid lithium-ion batteries have problems of volatilization, fire, and explosion by leakage of electrolyte because they use a solvent [4,5,6,7]. To solve this problem, many researchers have tried to design solid or semi-polymer electrolytes for little or no solvent [8,9]. Polymer electrolytes are known to be ideal because these electrolytes have high stability and flexibility, although their low conductivity needs to be improved. In addition, polymer electrolytes have excellent performances, such as machinability, chemical stability, flexibility, lightweight, and so on [10,11,12,13,14,15,16]. Nevertheless, it has been difficult to fabricate a polymer electrolyte membrane with adequate interfacial resistance because of its free volume and low adhesion between electrolyte and electrode in the process of growing a solid electrolyte interphase (SEI) [17,18].

For this reason, many researchers have investigated whether the ionic conductivity of a polymer electrolyte with a low glass transition temperature (T_g_) can be improved by increasing micro-Brownian motion and by reducing interfacial resistance. Micro-Brownian motion above a temperature of T_g_ can increase amorphous polymer chains and raise the adhesion when the polymer burrows into the electrodes [19]. For instance, Nair et al. [20] have demonstrated that a plasticizer can enhance the ionic conductivity of electrolytes based on poly (ethylene oxide) (PEO) and lead to good electrochemical stability and cycling performance. Liang et al. [21] reported that PEO-based electrolytes with succinonitrile (SN) as a plasticizer and dual-lithium-salt (LiBOB/LiTFSI) show outstanding stability in a cell as well. These plasticizers lead polymer electrolytes to have high ionic conductivity with flexibility because these plasticizers can lower the T_g_ of the electrolytes when they are mixed with polymer electrolytes.

To reduce the interface resistance, a strategy of using inner polymerization in a battery cell by a radical or thermal method has been proposed. The radical method used UV-curing with a photo-initiator on electrodes to induce direct polymer coating. This polymer electrolyte, after inner polymerization, shows good electrochemical performance and interfacial properties with a reduced free volume between the electrolyte and the electrode [22,23,24]. Ring-opening polymerization by cyclic compounds with Lewis acid as a thermal method has also been reported. Akbulut et al. [25] noted ring-opening polymerization of tetrahydrofuran (THF) with BF_3_ as an initiator. The polymer showed high ionic conductivity (>10^−4^ S/cm) at room temperature because of weak coordination between the lithium-ion and oxygen segments in the polymer main chain. Cui et al. [26] also reported that epoxy monomer with BF_3_ as an initiator can easily react and that the polymer has high ionic conductivity. In addition, Liu et al. [27] and Wu et al. [28] tried to synthesize polymer electrolytes by 1,3-dioxane and 1,3,5-trioxane with Li salts as an initiator, resulting in polymerization without the Lewis acid catalysts.

In this study, we attempt to utilize lithium bis(fluorosulfonyl)imide (LiFSI) as an initiator to carry out an in situ cationic ring-opening polymerization with a diepoxy monomer. A coin cell assembled with a LiFePO_4_/polymer electrolyte with 1.5 M LiFSI/Li had a capacity of 131 mAh/g at 0.2 C. Compared with commercial LiPF_6_, LiFSI has been extensively studied as a promising alternative conducting salt for LIBs. It exhibits not only superior stability and higher ionic conductivity but can also enhance electrochemical cyclability with graphite or Li metal anode [29,30,31]. The prepared polymer electrolytes exhibit good thermostability, better interfacial compatibility among components in the cell, and comparable conductivity (>0.1 mS/cm). The prepared polymer electrolyte also exhibited a comparable electrochemical window (ca. 4.0 V vs. Li/Li^+^), favorable compatibility with the electrode, and comparable cycling performances. These results indicated that the synthesized gel polymer electrolyte in the present work is a satisfactory candidate to improve the polymer electrolyte performance of LIBs.

## 2. Materials and Methods

### 2.1. Materials

LiFePO_4_-coated Al foil and lithium metal were obtained from the MTI corporation (Seoul, Korea). Neopentyl glycol diglycidyl ether (97%) (NGDE) and high-purity LiFSI (99.9%) were purchased from Sigma Aldrich (St. Louis, MO, USA) and used without further purification.

### 2.2. Preparation of Gel Polymer Electrolyte

The electrolyte solution was prepared with monomer NGDE and varied concentrations of LiFSI (0.5 M, 1 M, 1.5 M, and 2 M) and handled in an Ar-filled environment glovebox (H_2_O and O_2_ < 0.1 ppm). The obtained homogenous solutions were placed at 60 °C for 24 h, after completing polymerization. Finally, the received products were denoted as PNDGE 0.5, PNDGE 1, PNDGE 1.5, and PNDGE 2, respectively.

### 2.3. Characterization and Measurements

The structural characteristics of the polymer electrolytes were analyzed by Fourier-transform infrared spectra (FT-IR) with the use of a Nicolet iS5 (Thermo Fisher Scientific, Waltham, MA, USA), in the range 4000–600 cm^−1^, with a spectral resolution of 4 cm^−1^ at room temperature. Thermal properties were measured with a Scinco TGA-N 1000 (Seoul, Korea) analyzer from 30 °C to 500 °C at a heating rate of 10 °C/min in the presence of a N_2_ atmosphere. Differential scanning calorimetry (DSC) was conducted on DSC 6000 (Perkin-Elmer, Akron, OH, USA) from −60 to 75 °C, with a heating rate of 10 °C/min under a nitrogen atmosphere.

All the electrochemical impedance spectroscopy (EIS) was performed with an IM6ex, Zahner-Elektrik GmbH & Co. KG instrument (Kronach, Germany) for measuring the ionic conductivity (σ) of as-prepared electrolyte, under a temperature range of 30 to 80 °C at an interval of 10 °C, the frequency range of 0.1 Hz to 10^5^ Hz at the open-circuit potential, and an AC amplitude of 5 mV. The cells were allowed to reach a thermal equilibrium for 40 min before each test. Then, the data of the EIS spectra were fitted with an equivalent circuit model using Z-view software (version 3.1, Scribner Associates Inc., Southern Pines, NC, USA). The value of σ was calculated according to the following equation:

σ = L/(RA)(1)

where σ is the ionic conductivity, L is the distance between the two electrodes, R is the bulk resistance, and A is the active area of the electrode surface in contact with the electrolytes.

The cells were assembled inside a glovebox and all the electrochemical performances of the cells were observed using Ivium-n-Stat (Ivium Technologies B.V., Eindhoven, the Netherlands). The electrolyte was assembled based on the LFP/GPEs/Li cells at room temperature and was cycled between 2.0 and 4.0 V at room temperature. The C rate was defined based on the active cathode material, which was 12 mg/cm^2^. The cross-section of the electrolyte and electrodes in the cell was analyzed on JEOL 7401 F with an accelerating voltage of 15.0 kV.

### 2.4. Fabrication of Asymmetry Dummy Cell and Coin Cell

**Scheme 1:**
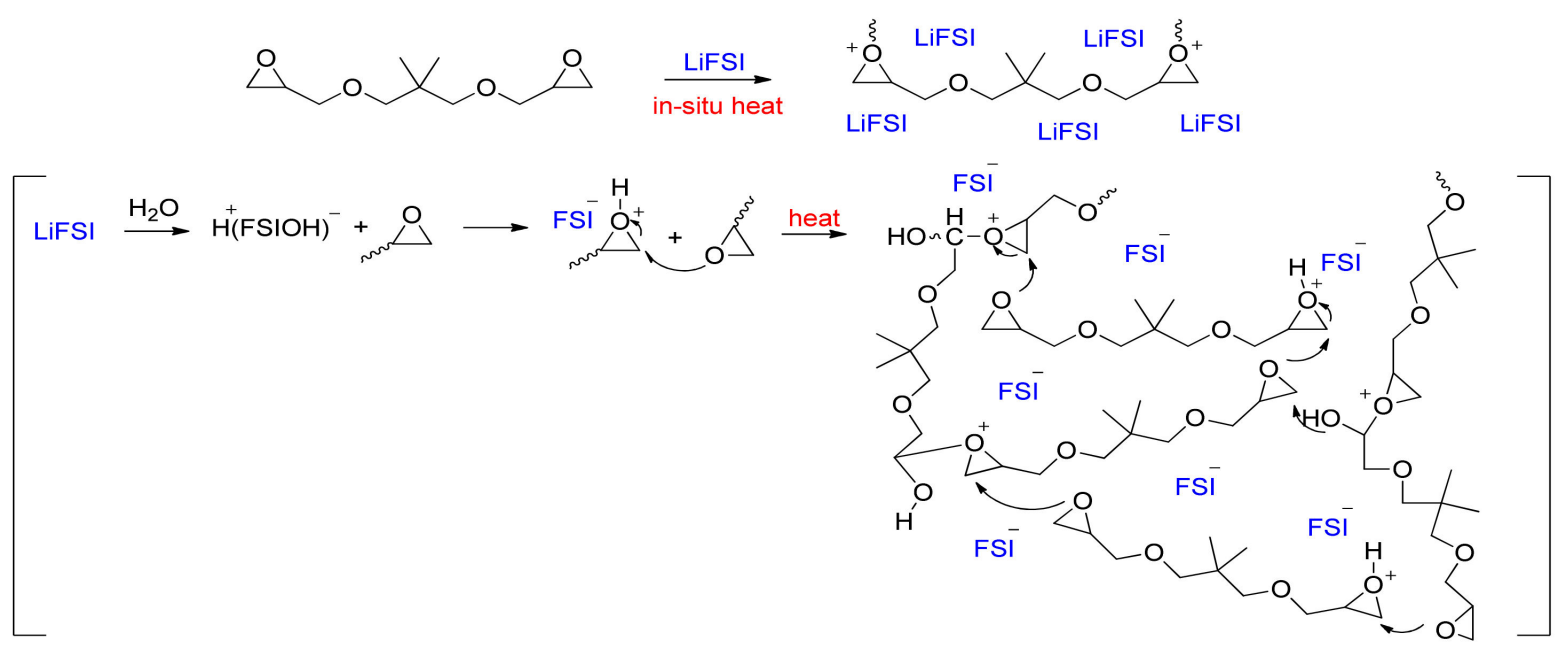
In situ polymerization steps for the synthesis of PNGDE.

Prior to evaluating the ionic conductivities of the as-prepared electrolytes, an asymmetric dummy cell was assembled with sealing tapes, electrolyte solutions, and FTO glasses, as shown in Figure S1. To prepare the FTO glasses, two glasses were cut to 2 cm × 3 cm (width × length), and one glass as the cathode electrode was drilled with two holes for the injecting of the electrolyte solution. Then, the FTO glasses were washed twice in methanol for 30 min to remove the rest of the glass fragments and dried at 60 °C overnight in a vacuum oven. The dummy cell with the PNGDE electrolytes for ionic conductivity were prepared in an Ar-filled environment glovebox. The clear electrolyte solutions were mixed with NGDE and various concentrations of LiFSI before the in situ polymerization and a plausible mechanism of ring-opening polymerization, which is illustrated in Scheme 1. The prepared clear solution was injected into the dummy cell through the pre-drilled hole on the FTO glass electrode and sealed by using Scotch tape. At the same time, the homogenous liquid solution was placed at a constant temperature of 60 °C for 24 h. In Figure S2, the electrolytes were carried out in a thermal-induced polymerization test with each molar concentration of LiFSI (0.5, 1, 1.5, and 2 M), to check the viscosity degree at 60 °C for 6, 12, 18, and 24 h, respectively.

The coin cell with PNGDE electrolytes for the electrochemical performance was also prepared in an Ar-filled environment glovebox, and the fabrication procedure of the cell is illustrated in Figure S3. The prepared electrolytes were poured in a silicone mold (5 cm × 5 cm) on a coated LiFePO_4_ electrode and heated at 60 °C for 24 h. After that, the coated electrode was punched to a suitable size (12 mm) for the coin cell. Finally, the coin cell was fabricated with a covering of lithium foil as an anode on the coated electrode.

## 3. Results

### 3.1. Characterization

**Figure 1:**
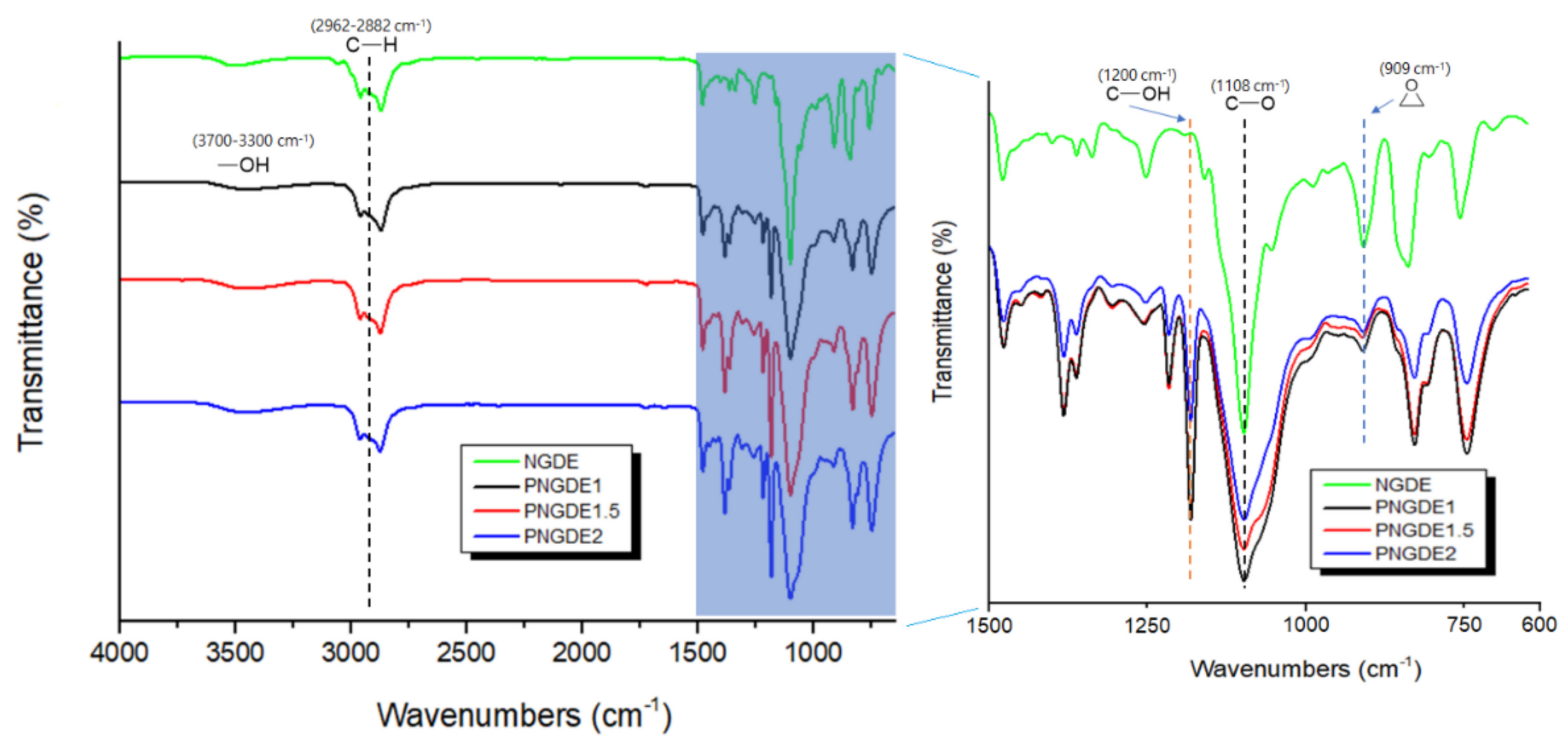
FT-IR spectra of NGDE monomer and PNGDE 1, PNGDE 1.5, and PNGDE 2 with enlarged scale of 1500–600 cm^−1^.

Figure 1 exhibits the FT-IR spectra of the NGDE monomer and the series of PNGDEs. All the materials displayed the common stretching vibration and bending vibration peaks of -C-O-C- (epoxy) and C-O (ether) at 909 cm^−1^ and at 1108 cm^−1^, respectively. They also displayed the main peaks of -CH_3_ and -CH_2_ (*sp*^3^), stretching at 2962 and 2882 cm^−1^, respectively. In addition, a peak at 840 cm^−1^, attributed to the Li-O rocking vibration, was observed, indicating an interaction of the ethylene oxide units with the Li ions [32]. The peak of epoxy at 909 cm^−1^ gradually disappeared by a degree of ring-opening polymerization of each of the PNGDEs with the increasing LiFSI ratio. On the other hand, the peak of a hydroxy group (-OH) and carbon-oxygen (C-OH) appeared at 3500 and 1200 cm^−1^, respectively. These peaks imply the reaction of the epoxy group via ring-opening polymerization. Thus, we calculated the conversion rate of the polymer electrolyte, which could be measured by the peak area of the epoxy bond (ca. 909 cm^−1^) at each PNGDE, using the following equation [33]:

Conversion rate = (A_0_ − A_t_)/(A_0_) × 100% (2)

where A_0_ and A_t_ are peak areas of the functional groups before and after polymerization, respectively. The conversion rates of the synthesized GPEs (PNGDE 1, PNGDE 1.5, and PNGDE 2) reached values at ca. 66.5%, 73.5%, and 76.1%, respectively. These results indicate the presence of GPEs along with the unreacted NGDE monomer with a probable need to suit a mole ratio of Li to O in the polymerization process [34].

### 3.2. Physicochemical Properties

**Figure 2:**
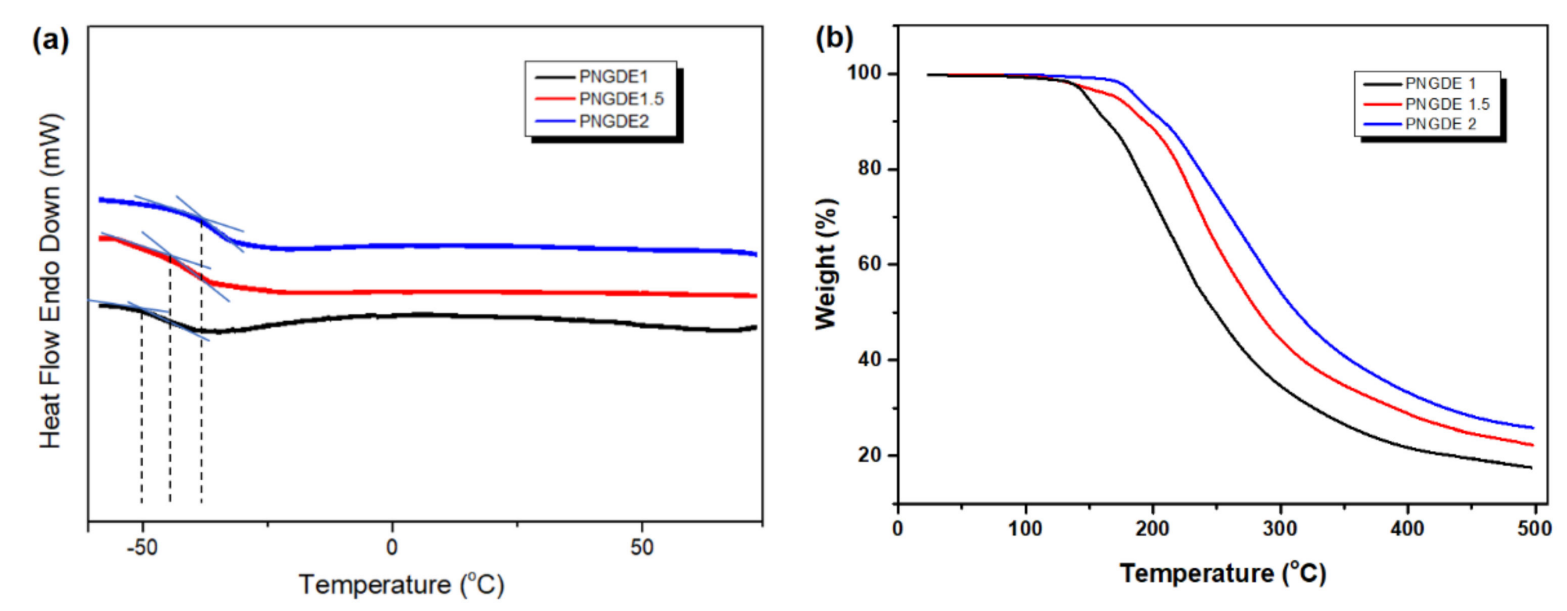
DSC curves (**a**) and TGA plots (**b**) of PNGDEs.

The glass transition temperature of PNGDE was measured by DSC from −55 to 80 °C. Figure 2a shows the peaks of T_g_ at −51, −43, and −38 °C for PNGDE 1, PNGDE 1.5, and PNGDE 2, respectively. The low T_g_ can provide electrolytes with excellent chain flexibility and increase the free volume for conducting lithium ions during charging and discharging, which is useful for improving the ionic conductivity of the electrolytes [35]. Figure 2b shows the thermo-gravimetric analysis (TGA) curves of the PNGDEs from 25 to 500 °C. All the GPEs exhibited two steps of weight loss. The initial 5% weight loss at around 100 °C was ascribed to the loss of a water by highly hydroscopic LiFSI [36]. The second weight loss was sharp up to 500 °C. The residual weight was probably ascribed to residual carbon compounds by thermal decomposition. Residuals need to further complete combustion over 500 °C. The residual percentage of PNGDE 2 was higher than that of PNGDE 1 or PNGDE 1.5 because of its higher concentration of lithium salt. These results imply that in situ polymer electrolytes can offer promising thermal stability, which satisfies the operation range of LIB.

### 3.3. Characterization of Interface on Electrolyte/Electrodes

**Figure 3:**
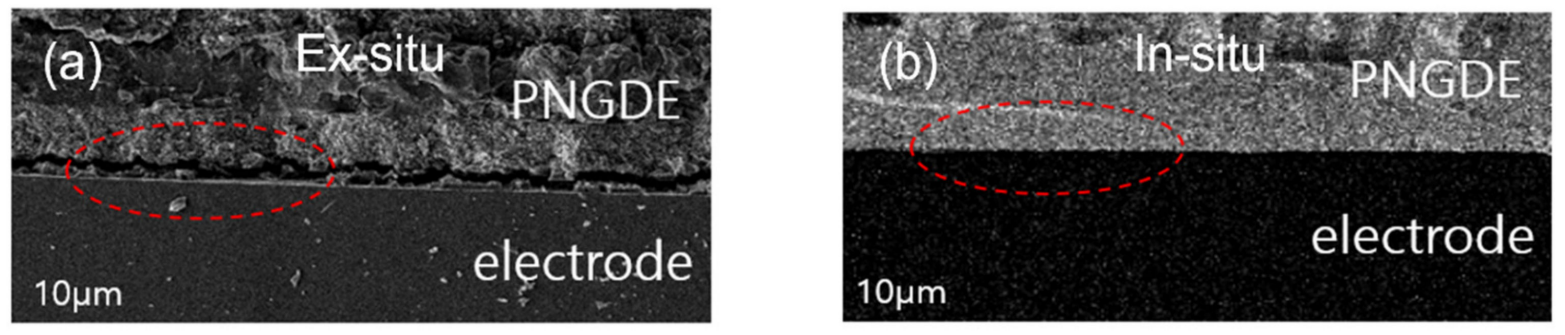
Cross-sectional FE-SEM images of interface via ex situ (**a**) and in situ (**b**) between PNGDE 1.5 and LiFePO_4_ (marked red dash line displayed corresponding interphase condition).

To confirm the interfacial contact between the synthesized electrolyte and the electrode, images of ex situ and in situ polymerized electrolytes were compared by field emission-scanning electron microscopy (FE-SEM) analysis. Figure 3a shows the interface between the electrolyte and the electrode fabricated by the ex situ method. Some empty spaces were observed between the electrolyte and the electrodes, resulting in a low ionic conductivity. On the other hand, Figure 3b shows a clear cross-section of the cell, in which the in situ polymerized electrolyte has a good contact with the electrode. Gaping holes at the interfacial contact area can induce low ionic conduction by interrupting the ion transfer between the electrolyte and the electrodes. This FE-SEM result confirms that the in situ polymer electrolyte is beneficial in improving the interface compatibility and in facilitating the ionic conductivity of polymer electrolytes as compared with the ex situ method [15,37].

### 3.4. Ionic Conductivity and Electrochemical Property

**Figure 4:**
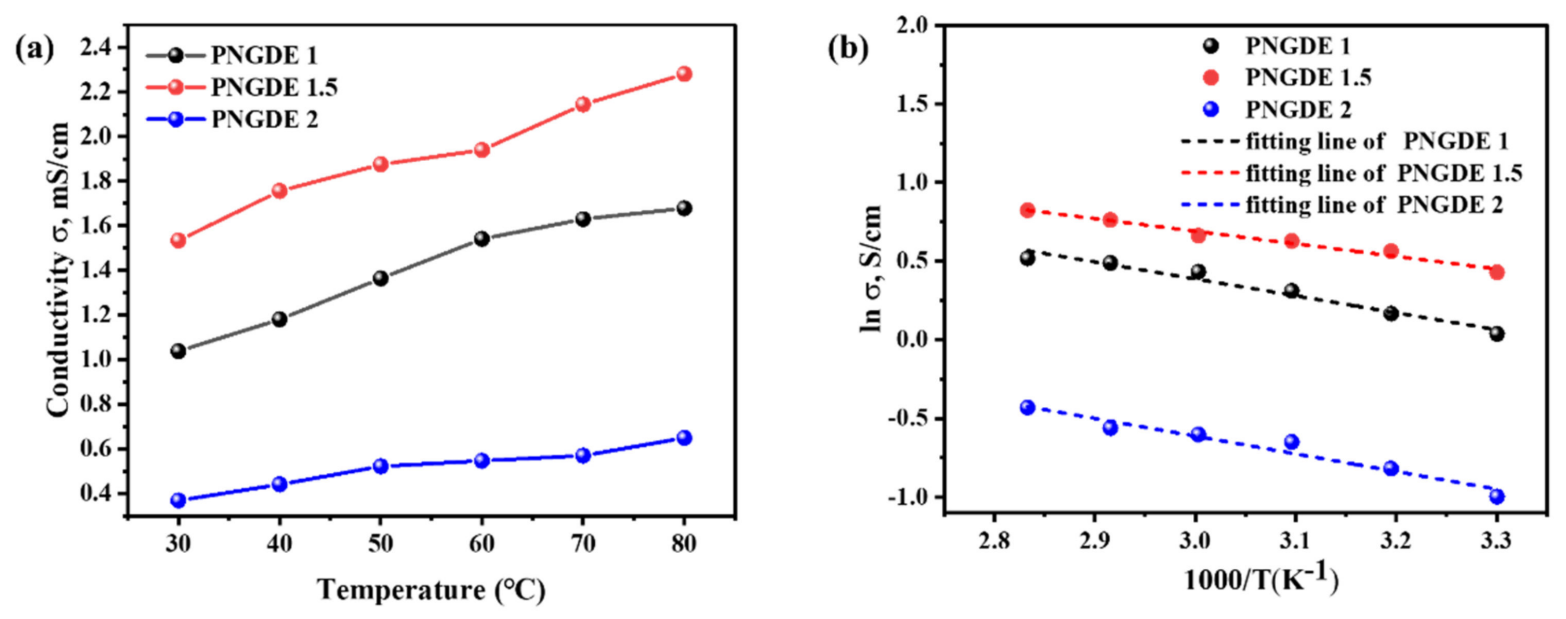
(**a**) Ionic conductivity versus temperature plots of PNGDEs and (**b**) Arrhenius plots of corresponding PNGDEs.

The temperature dependence of ionic conductivity for a series of PNGDEs was calculated from EIS plots, and all the EIS results were fitted with an appropriate equivalent circuit model via Z-view [38]. The ionic conductivities of the PNGDEs are present in Figure 4a, and the graph exhibited the ionic conductivities in a gradually elevated temperature range of 30–80 °C. The series of electrolytes show high conductivity of the range (6.23 × 10^−4^–2.25 × 10^−3^ S/cm) with the gradually elevated temperature because the flexibility of polymer is affected by T_g_, which is increased by the movement of the polymer at a high temperature [15]. The ionic conductivities of PNGDE 1, PNGDE 1.5, and PNGDE 2 were 3.62 × 10^−4^, 1.57 × 10^−3^, and 1.03 × 10^−3^ S/cm at room temperature, respectively. These results are attributed to the good contact between electrodes and electrolytes by the in situ method, which reduced the interfacial resistance [39]. In particular, the PNGDE 1.5 has higher ionic conductivity than the others at room temperature and extended to 80 °C (2.25 × 10^−3^ S/cm), which indicated that a suitable concentration of Li salt enhanced the conductivity of the electrolyte [40,41]. On the other hand, the PNGDE 2 shows as being lower than PNGDE 1.5 because a high Li salt concentration could lead to an ion aggregation and interrupt the polymerization of the epoxy group, resulting in polymer matrix pores of a reduced size. Furthermore, the ionic conductivity was studied by the Arrhenius plot, as shown in Figure 4b. The apparent activation energy (E_a_) of the ionic conductivity indicates the specific energy for migrating the active material through the polymer electrolyte. The E_a_ follows Arrhenius’s relationship with the gas constant (8.314 J/K mol) by the following Equation (3) [42]:

E_a_ = k_L_ × R (3)

where k_L_ is the slope of the line from the curve between lnσ and 1000/T. The E_a_ of PNGDE 1, 1.5, and 2 is calculated as 9.0 kJ/mol, 6.7 kJ/mol, and 9.4 kJ/mol, respectively. The lithium-ion migration in the polymer electrolyte is affected by E_a_ such that larger E_a_ values form large migration barriers for the active material. In addition, the higher value of E_a_ indicates that the ionic conductivity is temperature-sensitive, which is attributed to the in-creased movement of the polymer chain at elevated temperatures [42]. However, the PNGDE electrolytes exhibited low E_a_ values because they resulted in the remaining epoxy monomer, as shown in the conversion rate.

CV (as shown in Figure S5) was investigated with a potential window from −1.5 to 5.0 V, at a scanning rate of 1 mV/s at room temperature. Clearly exhibited is that the peaks of CV at 3.0 V and 2.1 V versus Li/Li^+^ were typical redox peaks, which could be ascribed to the delithiation and lithiumation of LiFePO_4_, respectively. In addition, the CV curves show overlapping with each other for five cycles and indicate the normal reversibility of the electrochemical reaction within the voltage range.

**Figure 5:**
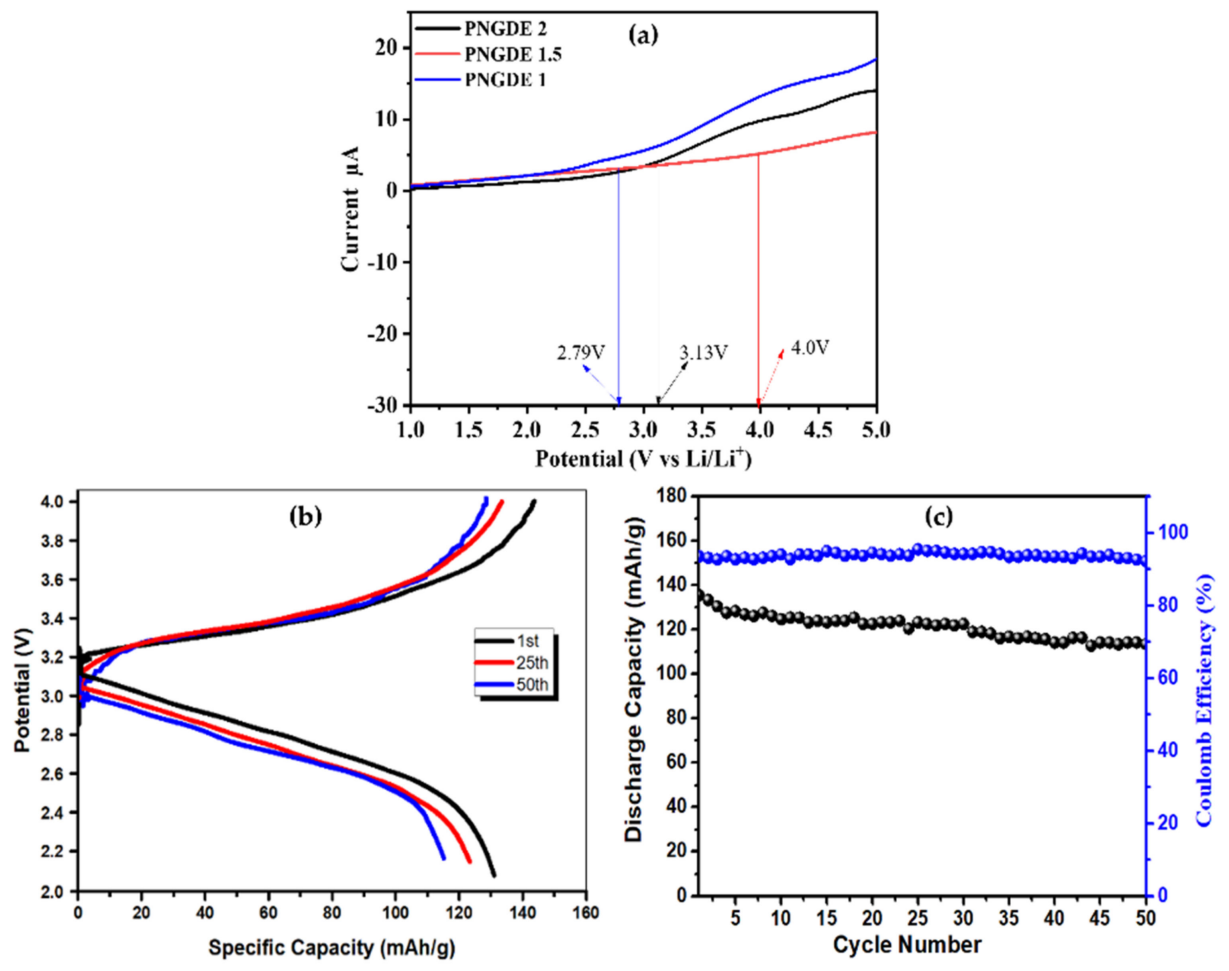
(**a**) LSV profiles of PNGDEs at scanning rate 0.1 mV/s from 1.0–5.0 V. (**b**) CD plots of the LiFePO_4_/PNGDE 1.5/LFP cell at 0.2 C. (**c**) Cycling performance of the LiFePO_4_/PNGDE 1.5/Li cell at 0.2 C after 50 cycles.

Figure 5 shows the electrochemical performances of the coin cell LiFePO_4_/PNGDE 1.5/Li, using GPE prepared through in situ polymerization technology. A linear sweep voltammogram (LSV) was carried out in the potential range from −1.0 to 5.0 V (vs. Li/Li^+^) at a scanning rate of 0.1 mV/s at room temperature. Figure 5a shows an oxidative decomposition before 4.0 V, suggesting that the in situ polymer electrolyte PNGDE 1.5 is stable up to 4.0 V. For other the polymer electrolytes, it was difficult to perform the cell test because of their narrow electrochemical stability window [43].

Figure 5b demonstrates the CD profiles of LiFePO_4_/PNGDE 1.5/Li at the 1st, 25th, and 50th cycle numbers over the potential range of 2.0–4.0 V at 0.2 C. The discharge-specific capacities of PNGDE1.5 after the 1st, 25th, and 50th cycles were 131, 123, and 115 mAh/g, respectively. The corresponding charge-specific capacities of PNGDE 1.5 were at 143, 133, and 127 mAh/g, respectively. In Figure 5c, the charge and discharge diagrams of the LiFePO_4_/PNGDE 1.5/Li coin cell were measured at an applied current of 0.2 C. The PNGDE 1.5 showed a capacity of up to 131 mAh/g at the first discharge. Its capacity then dropped to 115 mAh/g after 50 cycles, which had charge and discharge platforms, indicating that the cell conducted a reversible electrochemical reaction with a high degree of polarization. The long-term cycle stability indicates that the LiFePO_4_/PNGDE 1.5/Li-based asymmetrical coin cell shows a reasonable cycling performance, with the coulombic efficiency of 92%. However, this cell has a capacity lower than that of liquid electrolyte, although the interfacial resistance between the electrolyte and the electrodes reduced [39].

## 4. Conclusions

In this study, we synthesized a series of polymer electrolytes via in situ cationic polymerization with the di-epoxy group and LiFSI as an initiator. The synthetic method is simple for assembling the cell and shows good interfacial contact between the electrolyte and the electrodes. The in situ polymer electrolytes show good thermal stability (120 °C) and low T_g_ (−43 °C), indicating the obtainment of high ionic conductivity at 25 °C. In addition, the polymer electrolytes exhibit considerable ionic conductivity without organic solvent and compatible electrochemical stability. Finally, the first discharge capacity of the Li/PNGDE 1.5/LiFePO_4_ coin cell is 131 mAh/g, with the coulombic efficiency of 92% at 0.2 C. Although the PNGDE electrolytes did not show excellent long-term stability, the strategy of an in situ polymer can be a promising candidate for a lithium-ion battery in the electrolytes field.

## Data Availability

Not applicable.

## Supplementary Materials

The following supporting information can be downloaded at: https://www.mdpi.com/article/10.3390/membranes12040439/s1, Table S1: Comparison of properties of polymer electrolyte reported based on ring-opening polymerization; Figure S1: fabrication of asymmetry dummy ccell for measuring ionic conductivity; Figure S2: Photographing of various concentrations LiFSI with PNGDE up to 24 h; Figure S3: Fabrication and in situ process of coin cell without separator; Figure S4: Nyquist curves of PNGDE 1 (a), PNGDE 1.5 with fitting plots (b) and PNGDE 2 (c); equivalent circuit (d); Figure S5: Cyclic voltammetry profiles of PNGDE 1.5 over potential range from −1.5 to 5.0 V, scanning rate of 1 mV/s at room temperature. References [44,45,46,47,48,49] are cited in the supplementary materials.
