# Supplementary material for: Synthesis and Characterization of Gel Polymer Electrolyte Based on Epoxy Group via Cationic Ring-Open Polymerization for Lithium-Ion Battery
Source: Membranes (Basel). 2022 Apr 18;12(4):439. doi: 10.3390/membranes12040439 (PMC9031558; doi:10.3390/membranes12040439)
Supplement: Supplementary file 1 [file membranes-12-00439-s001.zip › membranes-1692979-supplementary.pdf]

Supporting information for

Synthesis and Characterization of Gel Polymer Electrolyte Based on Epoxy Group via Cationic Ring-Open Polymerization for Lithium-Ion Battery

Table S1. Comparison of properties of polymer electrolyte reported based on ring-opening polymerization

Components	Salt/Pasticizer/Solvent	σ(mS/cm) [a]	Stability vs (Li^+/Li) (V) and cathode	Ref.
PEO	LiTFSI/no/ACN	0.0004	4.8	1
DGEPEG, PEGDA	LiTFSI/no/no	0.053	4.7, LFP	2
POSS, P(EO-co-PO)	LiTFSI/ no/THF	0.11	5.4, LFP	3
GLYMO, EDGE	LiTFSI/no/Ethanol	0.026	4.9, LTO	4
GLYMO, DGEPEG	$LiClO_4$/no/no	0.12	N/A	5
BDE, ED600	LiTFSI	0.5 [b]	4.51, LFP	6
PNGDE-1.5	LiFSI/no/no	1.57	4.0, LFP	This work

1) [a] at 25 °C unless notes

2) [b] at 45 °C

2) Acetonitrile (ACN), tetrahydrofuran (THF), lithium bis(trimethanesulfonyl)imide (LiTFSI), $LiFePO_4$ (LFP), $Ni_{1/3}Mn_{1/3}Co_{1/3}O_2$ -NMC, $Li_4Ti_5O_{12}$ (LTO), Poly (ethylene oxide) (PEO), Diglycidylether of polyethylene glycol (DGEPEG), Poly (ethylene glycol) diacrylate (PEGDA), Polyhedral oligomeric silsequioxane (POSS), Poly(ethylene-co-propylene oxide) (P(EO-co-PO)), (3-glycidyloxypropyl) trimethoxy silane (GLYMO), Ethyl glycol diglycidyl ether (EDGE), Bisphenol A diglycidyl ether (BDE), O,O-bis(2-aminopropyl) polypropylene glycol-*block*-polypropylene glycol (ED600)

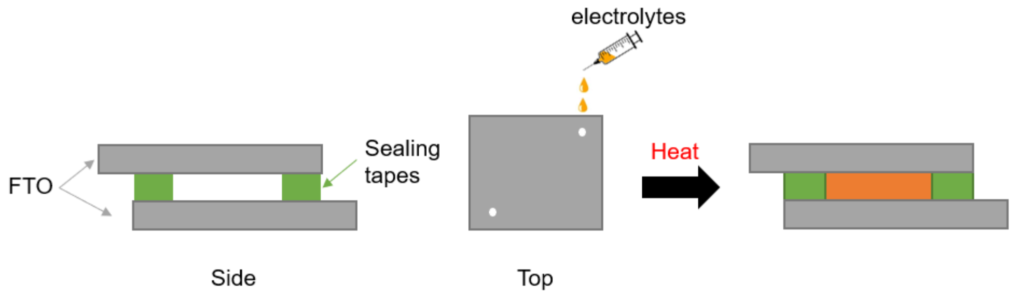

Figure S1. Fabrication of asymmetry dummy cell for measuring ionic conductivity.

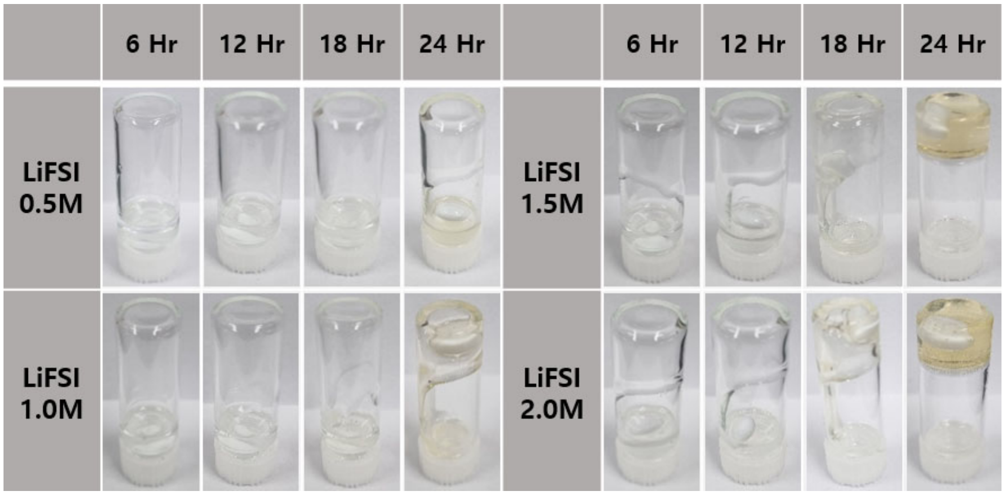

Figure S2. Photographing of various concentrations LiFSI with PNGDE up to 24 h.

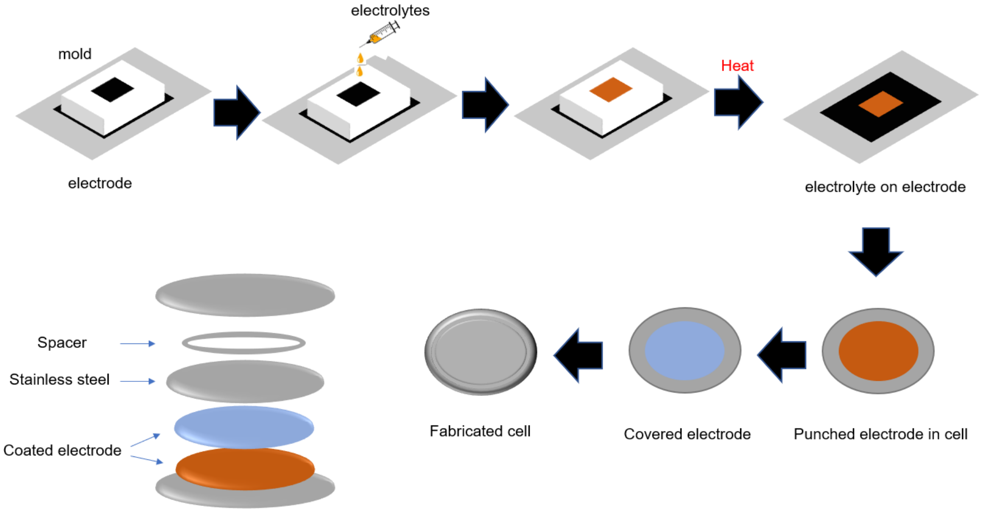

Figure S3. Fabrication and in situ process of coin cell without separator.

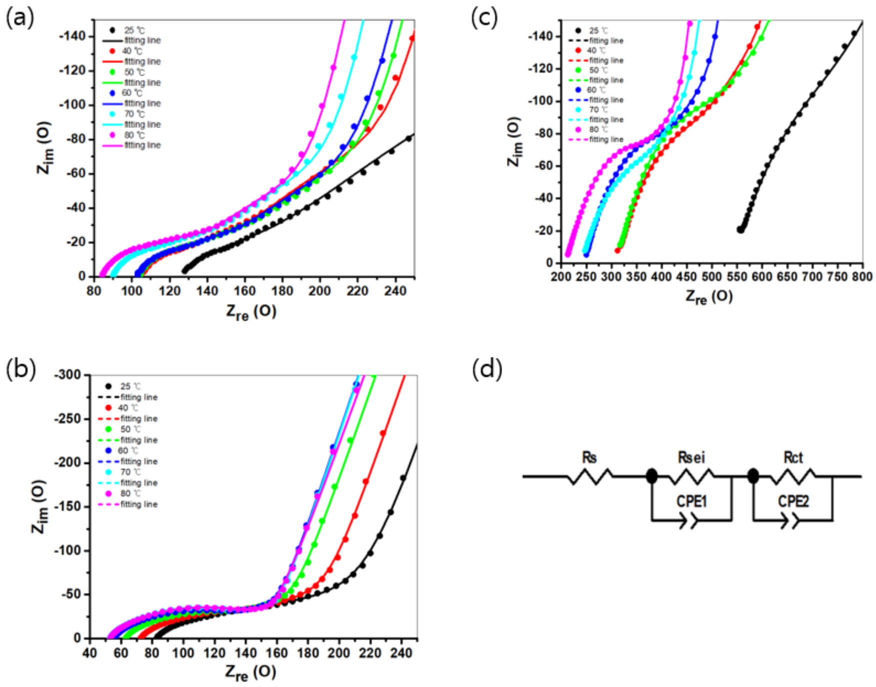

Figure S4. Nyquist curves of PNGDE1 (**a**), PNGDE1.5 with fitting plots (**b**) and PNGDE2 (**c**); equivalent circuit (**d**).

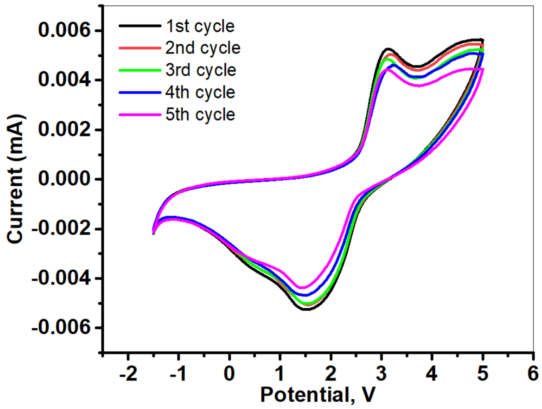

Figure S5. Cyclic voltammetry profiles of PNGDE 1.5 over potential range from -1.5 to 5.0 V, scanning rate of 1 mV/s at room temperature.

References

1. Cheng, H.; Zhu, C.; Huang, B.; Lu, M.; Yang, Y. Synthesis and electrochemical characterization of PEO-based polymer electrolytes with room temperature ionic liquids. *Electrochim. Acta* **2007**, *52*, 5789–5794.

doi: 10.1016/j.electacta.2007.02.062.

2. Duan, H.; Yin, Y.X.; Zeng, X.X.; Li, J.Y.; Shi, J.L.; Shi, Y.; Wen, R.; Guo, Y.G.; Wan, L.J. In situ plasticized polymer electrolyte with double-network for flexible solid-state lithium-metal batteries. *Energy Storage Mater.* **2018**, *10*, 85–91. doi: 10.1016/j.ensm.2017.06.017.
3. Hsu, S.T.; Tran, B.T.; Subramani, R.; Nguyen, H.T.T.; Rajamani, A.; Lee, M.Y.; Hou, S.S.; Lee, Y.L.; Teng, H. Free-standing polymer electrolyte for all-solid-state lithium batteries operated at room temperature. *J. Power Sources* **2020**, *449*, 227518. doi: 10.1016/j.jpowsour.2019.227518.
4. Vélez, J.F.; Aparicio, M.; Mosa, J. Covalent silica-PEO-LiTFSI hybrid solid electrolytes via sol-gel for Li-ion battery applications. *Electrochim. Acta* **2016**, *213*, 831–841. doi: 10.1016/j.electacta.2016.07.146.
5. Popall, M.; Andrei, M.; Kappel, J.; Kron, J.; Olma, K.; Olsowski, B. ORMOCERs as inorganic-organic electrolytes for new solid state lithium batteries and supercapacitors. *Electrochim. Acta* **1998**, *43*, 1155–1161. doi: 10.1016/S0013-4686(97)10014-7.
6. Tian, G.; Zhao Z.; Zinkevich, T.; Elies, K.; Scheiba, F.; Ehrenberg, H. A crosslinked Polyethyleneglycol Solid Electrolyte Dissoving Lithium Bis(trifluoromethylsulfonyl)imide for Rechargeable Lithium Batteries. *ChemSuschem* **2019**, *12*, 4708-4718. doi: 10.1002/cssc.201901587.
